# Micellar Drug Delivery Systems Based on Natural Biopolymers

**DOI:** 10.3390/polym13030477

**Published:** 2021-02-02

**Authors:** Leonard Ionut Atanase

**Affiliations:** Department of Biomaterials, Faculty of Medical Dentistry, “Apollonia” University of Iasi, Pacurari Street, No. 11, 700511 Iasi, Romania; leonard.atanase@univapollonia.ro

**Keywords:** micelle, self-assembly, polysaccharides, peptides, proteins, drug delivery systems

## Abstract

The broad diversity of structures and the presence of numerous functional groups available for chemical modifications represent an enormous advantage for the development of safe, non-toxic, and cost-effective micellar drug delivery systems (DDS) based on natural biopolymers, such as polysaccharides, proteins, and peptides. Different drug-loading methods are used for the preparation of these micellar systems, but it appeared that dialysis is generally recommended, as it avoids the formation of large micellar aggregates. Moreover, the preparation method has an important influence on micellar size, morphology, and drug loading efficiency. The small size allows the passive accumulation of these micellar systems via the permeability and retention effect. Natural biopolymer-based micellar DDS are high-value biomaterials characterized by good compatibility, biodegradability, long blood circulation time, non-toxicity, non-immunogenicity, and high drug loading, and they are biodegraded to non-toxic products that are easily assimilated by the human body. Even if some recent studies reported better antitumoral effects for the micellar DDS based on polysaccharides than for commercial formulations, their clinical use is not yet generalized. This review is focused on the studies from the last decade concerning the preparation as well as the colloidal and biological characterization of micellar DDS based on natural biopolymers.

## 1. Introduction

Nanomedicine is an emerging science in which nanotechnology is applied in order to improve the healthcare by preparing drug nanocarriers that can passively or actively target the tumor tissues, due to an enhanced permeability and retention effect (EPR), thus leading to a more efficient treatment. In the last two decades, the design of a suitable drug carrier was the main focus in developing new medicines. An ideal drug carrier must be biocompatible, biodegradable, non-toxic, and have the capability to release the loaded active principles at the desired site of action. Until now, a large number of inorganic and organic materials, which have been formulated as liposomes, organic and inorganic nanoparticles, and hydrogels, have been used as promising drug carriers [1,2,3,4,5,6,7,8]. However, there is an increasing interest in using natural biopolymers for the elaboration of different types of drug delivery systems (DDS) due to their abundance in nature and characteristic features, such as sustainability, biocompatibility, and biodegradability [9,10].

**Figure 1:**
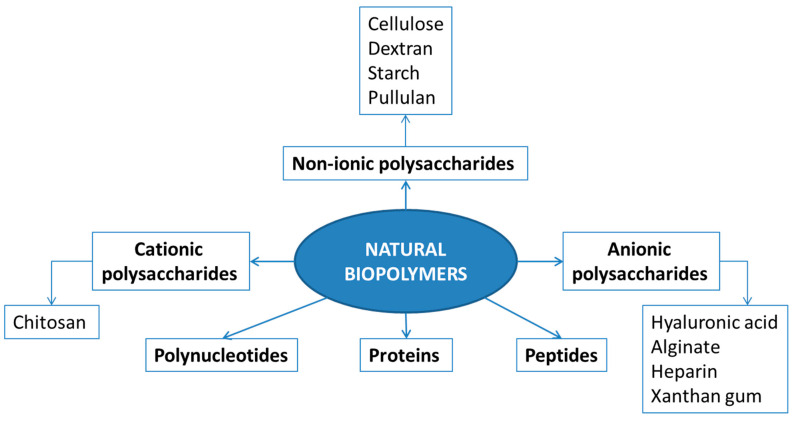
Different types of natural biopolymers.

Natural biopolymers are produced by the cells of living organisms and consist of monomeric units that are covalently bonded to yield high molecular weight molecules. The different types of natural biopolymers are illustrated in Figure 1.

The most frequently used types of natural biopolymers are polysaccharides and proteins, which are the main focus of this review. Polysaccharides, mainly obtained from algae, plants, microbes, and animals, may be classified as a function of their charge: cationic (e.g., chitosan), anionic (e.g., hyaluronic acid, alginate, heparin, and xanthan gum), and non-ionic (e.g., cellulose, dextran, and starch). Proteins are a versatile class of biopolymers consisting of one or more long chains of amino acid residues in their composition and having different physicochemical properties. A short linear chain containing less than 20–30 amino acid residues are commonly called peptides or oligopeptides.

Owing to the presence on their molecular chains of various functional groups, such as hydroxyl, carboxyl, and amino groups, these natural biopolymers can be easily chemically modified with side chains in order to overcome their limitations, such as low solubility in common solvents and batch-to-batch variation. Recent advances in synthetic biology and bioengineering methods allowed the preparation of polysaccharides or protein-based innovative amphiphilic copolymers used for specific application, as for example, micellar DDS [11].

Among all the existing DDS, polymeric micelles (PMs) are of particular interest because the hydrophobic drug loaded in micellar hydrophobic core is protected by the outer hydrophilic corona, which can further prevent micelles from being eliminated by the mononuclear phagocytic system (MPS) in physical circulation, thereby prolonging the in vivo circulation time. The driving force behind the micelle formation is the decrease in the free energy of the system due to the removal of hydrophobic segments from the aqueous environment to form the micellar core. An important consideration for drug delivery is the relative thermodynamic (potential for disassembly) and kinetic (rate of disassembly) stability of the PMs after the intravenous injection and subsequent extreme dilution in the vascular system. The thermodynamic stability of the micelles can be explained in terms of critical micelle concentration (C.M.C.). Increasing the hydrophobicity of an amphiphilic copolymer reduces the C.M.C., which in turn increases the micelle stability. The incorporation of hydrophobic drugs into the micellar core may also further enhance micelle stability. This is an important feature for injectable biomedical applications because PMs with lower C.M.C. values prevent the dissociation into unimers upon dilution with large volume of blood. In addition, PMs are intrinsically stealth particles, and they can avoid engulfment by immune-system cells without further modification. Moreover, the relatively small micellar size allows their passive accumulation via the EPR effect even in neovascularized or poorly vascularized tumors, which would lead to a reduced systemic toxicity [2,12].

**Figure 2:**
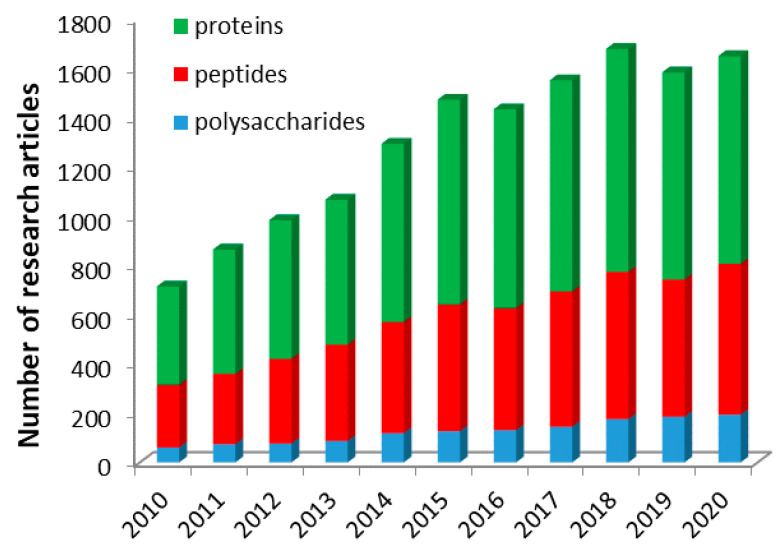
Number of research articles, from the last decade, given by Scopus using a combination of three keywords, such as: micelle, polysaccharide or peptides or proteins, and drug delivery system.

As the number of research articles from the last decade concerning the micellar drug delivery systems based on polysaccharide, peptide, and proteins is in a continuous increase, as illustrated in Figure 2, this review is focused on the most important studies from the last decade concerning the preparation as well as the colloidal and biological characterization of micellar DDS based only on graft and block amphiphilic copolymers of these natural biopolymers.

Even if the number of research studies given in Figure 2 may seem enormous, at the first view, in reality, the number of papers in which a model drug was loaded into the obtained micelles is much lower, and in this review, only the studies in which a drug was used were taken into account. Therefore, the studies related to the preparation and characterization of empty micelles are without the scope of this review.

## 2. Self-Assembly and Drug-Loading Methods

**Figure 3:**
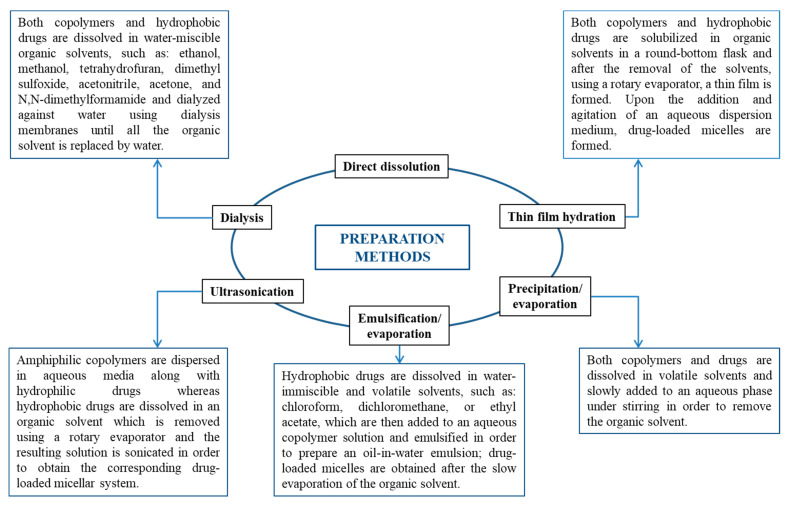
Preparation methods of both empty and drug-loaded micellar systems.

In addition to the molecular weight of the amphiphilic copolymers, the self-assembly method strongly influences both the micellar sizes and size distribution (polydispersity). The general preparation methods of nanocarriers based on biopolymers were reviewed recently by Samrot et al. [13]. Therefore, the self-assembly methods are only briefly presented in Figure 3.

Direct dissolution is the easiest method for the preparation of empty PMs, but this method can also lead to the formation of micellar aggregates in a large extent [11,14]. Dialysis is one of the usually used methods, as the system reaches a thermodynamic equilibrium avoiding, thus the micellar aggregation.

**Figure 4:**
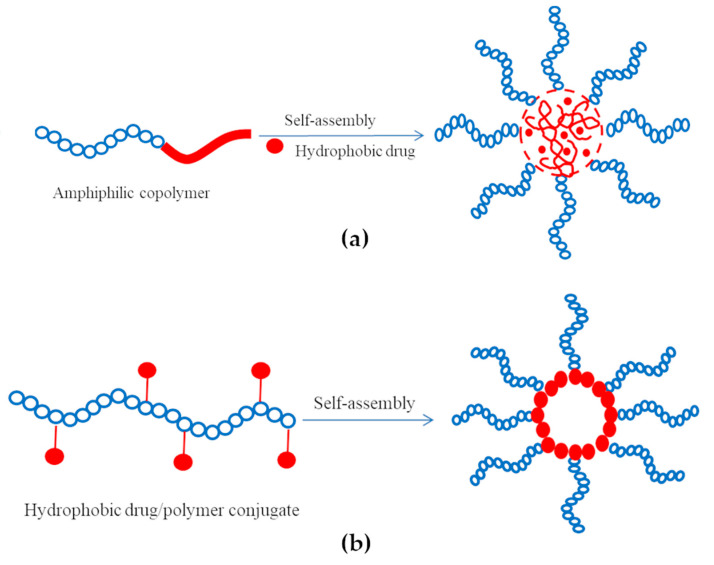
Schematic representation for the preparation of micellar drug delivery systems (DDS) by self-assembly; (**a**) physical drug entrapment; (**b**) self-assembly of drug/polymer conjugate formed by chemical or polyionic conjugation.

Drugs can be loaded into PMs using three different methods: physical entrapment of drug into the micellar core, polyionic complexation (e.g., ionic binding) between drug and micellar corona, and chemical conjugation of drugs. The schematic representation of the self-assembly process of amphiphilic copolymers for the preparation of drug-loaded micelles is given in Figure 4.

The physical entrapment of hydrophobic drugs is generally preferred over micelle-forming polymer-drug conjugates as the intrinsic characteristics (C.M.C. and aggregation number) of the latter system can be altered by the low content of hydrophobic part. Regardless of the preparation method, the drug can be released through polymer degradation, diffusion, or demicellization upon dilution [15].

The main characteristics of different micellar DDS based on drug/biopolymer conjugates are provided in Table 1.

The physical loading efficiencies of the drug molecules in polymeric micelles were found to be dependent on the incorporation methods. With the exception of direct dissolution, all the other methods given in Figure 3 can be used for the preparation of drug-loaded micellar systems. However, at the present, there seems not to be one universal method for the preparation of drug-loaded PMs with high encapsulation and loading efficiencies. Moreover, the micellar sizes and stability as well as drug encapsulation efficiency are highly dependent on the nature of the solvent used in the preparation method of these DDS. Furthermore, the preparation methods of these DDS could be difficult to scale up from a small laboratory scale to an industrial one. Therefore, based on a deep knowledge of the molecular structure of both drugs and amphiphilic copolymers, systematic studies are needed in order to establish and develop highly efficient encapsulation methods that can provide a sufficient concentration around the therapeutic dose of the drug for in vivo applications.

## 3. Characterization Methods of Drug-Loaded Micellar Systems

It is well accepted that a complete physicochemical characterization of the micellar systems will be of great advantage in explaining their in vitro behavior during the biological tests [35].

The characterization techniques of empty and drug-loaded micellar systems have been extensively reviewed in the literature and therefore, in this paragraph, they will be only briefly discussed [11,14]. Table 2 gives the most important colloidal characteristics of both empty and drug-loaded micellar systems as well as the corresponding characterization techniques.

*DLE*% and *DEE*% are the most important parameters characterizing the drug-loaded systems and can be determined using Equations (1) and (2).

(1) $$DLE\left(\%\right)=\;\frac{Amount\;of\;drug\;in\;micelles}{Amount\;of\;added\;polymer+drug}\times 100$$

(2) $$DEE\left(\%\right)\;=\;\frac{Amount\;of\;drug\;in\;micelles}{Amount\;of\;added\;drug}\;\times \;100$$

Micellar stability as a function of storage time, temperature, pH, and in different physiological environments must be taken into account in all studies as it is of prime importance, especially for drug-loaded micellar systems. Another important aspect of these systems concerns the micellar stability as a function of time or temperature. The evaluation of the zeta potential (ZP) values or of the micellar sizes is recommended in order to assess the stability. The stability during storage might be increased by freeze-drying of the micellar systems in the absence or in the presence of a cryo-protectant substance.

Finally, it is important to note that polydispersity in composition, molecular weight, and architecture of the amphiphilic copolymers based on biopolymers may have important effects on both drug entrapment efficiency and release kinetics.

## 4. Micellar Drug Delivery Systems Based on Polysaccharides

Polysaccharides are polymeric carbohydrates composed of repeating monomeric units of monosaccharides that are covalently linked to each other through glucosidic linkage. Polysaccharides are biocompatible, biodegradable biopolymers, and the presence of various functional groups, such as hydroxyl, carboxyl, and amino groups, allow their easy chemical modification in order to increase their intrinsic properties (solubility, chemical stability, etc.). Due to these improved properties, polysaccharides are largely used as biomaterials in food, biomedical, pharmaceutical, cosmetic industry, and also as micellar drug-loaded systems [11,36]. This section will review the micellar DDS based on polysaccharides in which the drug was entrapped by physical methods.

### 4.1. Drug Delivery Systems Based on Cationic Polysaccharides

**Figure 5:**
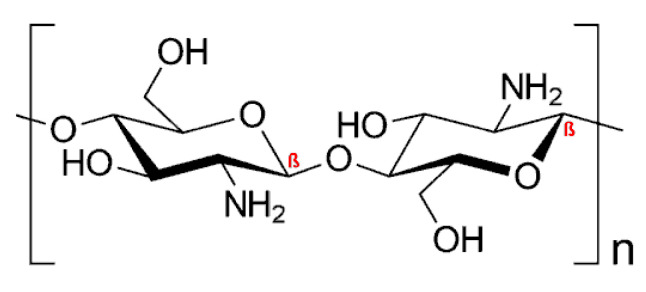
Chemical structure of chitosan (CS).

Chitosan (CS), the only naturally occurring cationic polymer, is renewable, inexpensive, and an environmentally friendly source of polycationic macromolecular building blocks owing to the chemical modification at the free amino (N-grafting) or hydroxyl (O-grafting) groups. The chemical structure of CS is provided in Figure 5:

CS was modified with succinic anhydride and octaldelhyde followed by the conjugation of folate, as a ligand, in order to obtain micellar system for the tumor active targeting [37]. Relatively stable micelles for 15 days in phosphate-buffered saline (PBS) (pH = 7.4) and having an average diameter of 200 nm were prepared by a precipitation method followed by dialysis. Micellar DDS were obtained using fluorescein and indocyanine green (ICG) derivatives as hydrophobic model molecules. Both DEE and DLE were higher for fluorescein than for ICG derivatives due to the higher solubility. In vivo tests have clearly demonstrated the enhanced tumor targeting effect of these folate-functionalized CS-based micelles. Folate was also used as a ligand, in a more recent study, for the preparation, by precipitation followed by dialysis, of *N-*naphtlyl-*N*,*O*-succinyl CS micelles loaded with a semi-synthetic andrographolide analogue 3A.1 (19-tert-butyldiphenylsilyl-8,17-epoxy andrographolide) [38]. The size of these drug-loaded micelles was smaller than 200 nm and a negative ZP value of −31.7 mV was determined, indicating a good stability. As previously indicated, the folate moiety induced a higher anticancer activity against HT29 cancer cells than the non-functionalized micelles.

Stearic acid (SA) was used for the preparation of a series of amphiphilic copolymers based on low molecular weight CS, which self-assembled in aqueous medium in micelles with average sizes in the range of 33.4 to 130.9 nm and ZP values of 22.9 to 48.4 mV [39]. A SA substitution degree (DS) of around 40% led to smallest micellar size, C.M.C. value, and highest ZP value. Doxorubicin (Dox) was used as a model drug for in vitro and in vivo tests of these drug-loaded micellar systems, prepared by dialysis, as oral DDS, and it appeared that CS-based PMs increased the bioavailability of drug by prolonging its circulation time. The same hydrophobic modification was also performed by Moazeni et al. [40], but in this case, the micelles were loaded with itraconazale as a model drug for pulmonary delivery. The drug-loaded micelles, prepared by a thin film hydration method, had sizes in the range of 120 to 200 nm, and both DEE and DLE values were low. Moreover, the ZP values ranged from 34 to 74 mV, which were much higher than those obtained by Yuan et al. [39]. The nebulization efficiency was up to 89%, and these PMs maintained the drug stability during the inhalation process.

Fattahi et al. [41] have used CS-graft-retinoic acid micelles as gene carriers. Size, zeta, and C.M.C. of these micelles were 142.14 ± 5.06 mg/mL, 27.25 ± 6.31 mg/mL, and 1.3 × 10^−2^ mg/mL, respectively. The cellular uptake of these micelles was more effective in Hela than in HepG2 cells. The obtained results confirmed the fact that these micellar systems are safe and effective nanocarriers for in vitro and potentially in vivo gene delivery.

Xu et al. [42] have conjugated prostate cancer-binding peptides (PCP) with hydrophobic cholesterol-modified glycol CS (CHGC) at two different DS. Dox-loaded micelles were prepared by an emulsion/evaporation method, and it was noticed that the drug loading led to an increase of the micellar size from 246 to 293 nm. Moreover, DLE and DEE values were 11.4% and 85.8%, respectively. In vitro cytotoxicity and cellular uptake showed an enhanced intracellular drug delivery and a higher tumor inhibition owing to the presence of PCP. In another study, Muddineti et al. [43] have also used cholesterol in order to obtain an amphiphilic graft copolymer based on CS. The dialysis method was used for the preparation of Cur-loaded micelles, and the average values for DEE and DLE were 35.9% and 35.0%, respectively. The micelles prepared from the optimum formulation, having CS and cholesterol amounts of 10 mg and 5.24 mg, had an average diameter of 162 nm. In vitro cytotoxicity tests were performed on both murine melanoma and human breast cancer cells and showed an enhanced cytotoxic effect compared to free Cur. More recently, the same group has loaded both Cur and siRNA in the same type of PMs [44]. In this case, the average micellar diameter was 165 nm, and a ZP value of 24.8 mV was determined. Another amphiphilic graft copolymer was synthesized by the modification of CS with hydrophobic oleic acid (OA) [45]. Micelles, with an average diameter of 520 nm and a ZP value of 42 mV, were obtained by a thin film hydration method and were loaded with cefixime trihydrate (CFX). The influence of pH on drug-loading efficiency was investigated, and it appeared that DLE was maximum (55%) at pH = 5.5. The in vitro release studies revealed 52% of drug release after 24 h of incubation and were enhanced to 83% after 72 h in simulated intestinal fluid condition.

Recently, gambogic acid was loaded in *O*,*N*-hydroxyethyl CS-graft-octylamine graft copolymer micelles [46]. The coating with hyaluronic acid (HA) of these micelles, obtained by a dialysis method, has not affected the micellar size or the loading capacity. However, a ZP inversion, from around 20 to −24 mV, was observed after the HA coating. The efficiency of these micellar systems as DDS was investigated by in vitro and in vivo tests, and it was demonstrated that the HA-coated drug-loaded micelles have a higher antitumoral efficiency than the non-coated ones.

Hydrophilic mPEG was used for the synthesis of an amphiphilic CS-graft-mPEG graft copolymer, with a DS of 3.23% [47]. Starting from this copolymer, spherical micelles, with an average diameter of 200 nm, were prepared by an ultrasonication method. The loading of 5-fluorouracil (5-FU), as a model antitumoral drug, led to an average diameter of 769 nm for a 5-FU/CS-graft-mPEG weight ratio of 5/10 (*w*/*w*). Zhao et al. [48] prepared a similar micellar DDS which was further loaded with Dox by a dialysis method. These drug-loaded micelles, with a core of 64 nm and a corona of 14 nm in PBS at pH = 7.4, were pH-sensitive due to the ability of CS to switch from hydrophilic to hydrophobic as the pH switched from pH < 5 to pH = 7.4.

**Figure 6:**
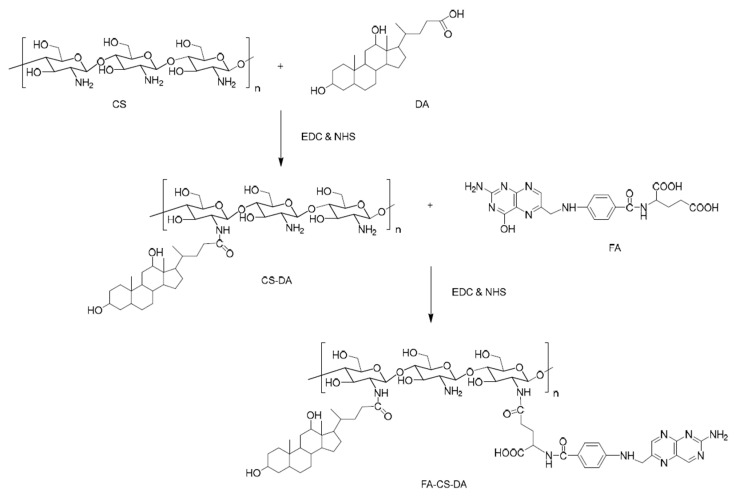
Schematic synthesis route of folic acid (FA)–CS–deoxycholic acid (DA) copolymer [52].

An interesting dual-targeting micellar system, based on succinyl-CS, folic acid (FA), and methianine (Met), was prepared by Chen et al. [49]. In addition, a fluorescence probe was conjugated as a hydrophobic group. Paclitaxel (PTX) was loaded in these spherical micelles with an average diameter of 200 nm. It was demonstrated that the active targeting ability of these micellar DDS was enhanced by the conjugations of both FA and Met as ligands. PTX was also loaded in micelles prepared, by dialysis, from similar FA–cholesterol–CS biopolymer [50]. The same drug, PTX, was used for the preparation of drug-loaded micelles based on an amphiphilic CS-graft-α-tocophenol succinate copolymer [51]. Spherical drug-loaded micelles were obtained by an ultrasonication method, and the loading process increased the average micellar diameter from 35 to 142 nm. Moreover, these PTX-loaded micelles proved in vitro and in vivo increased antitumoral activity. PTX-loaded micelles were also prepared from an amphiphilic copolymer, based on CS, after the previous hydrophobic modification of CS with two hydrophobic moieties, deoxycholic acid (DA) and FA at DS values of 15.8% and 8%, respectively [52]. The synthesis route of the amphiphilic graft copolymer based on CS is illustrated in Figure 6.

**Figure 7:**
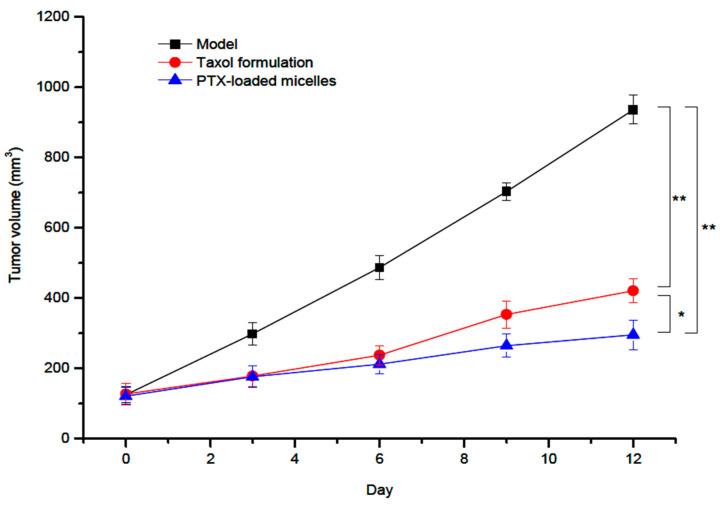
In vivo antitumor activity of Paclitaxel (PTX)-loaded mPEG-CS(SH)-CHO micelles: tumor volume growth curves after various treatments (* *p* < 0.05; ** *p* < 0.01) [55].

Spherical micelles, obtained by an ultrasonication method, were characterized by an average diameter of 126 nm and a positive ZP value of 19.3 mV. Moreover, the DLE and the DEE values of these PTX-loaded micelles were 9.1% and 81.2%, respectively. By performing in vitro and in vivo tests, it was concluded that these micellar systems are suitable carriers for the intravenous administration of PTX. More recently, a novel amphiphilic graft copolymer based on CS and *N*-octyl-*N*′-phthalyl-O-phosphoryl was synthesized and used for the loading and oral delivery of PTX [53]. The micellar system, with a mean diameter of 137 nm and a ZP value of 27.99 mV, was obtained by a dialysis method. In order to improve the stability in gastric fluids, this micellar system was filled into enteric-coated capsules. It have been demonstrated that this formulation increased the oral bioavailability of PTX for transcytosis across entorecytes. Another very recent study concerning the improvement of the oral bioavailability of PTX was carried out by Yang et al. [54]. The micellar DDS is based on L-carnitine (LC) conjugated CS–SA polymeric micelles, which were prepared by solvent evaporation–hydration method. The ligand LC was conjugated onto the micelle surface by anchoring its derivative stearoyl group to the lipophilic SA micellar core. The PTX-loaded micelles showed regular spherical shapes with relatively small average diameters of 157.1 nm and high DLE of 15.96%. Moreover, the micellar stability in water was supported by a low C.M.C. value of 14.31 ± 0.21 μg/mL. Furthermore, these drug-loaded micelles presented a slow and incomplete in vitro PTX release, and the pharmacokinetic studies indicated an increase of the relative bioavailability of PTX to 165.8% against Taxol, which is a commercial formulation. In another very recent study, a comparison between the efficiency of the commercial formulation Taxol and that of a redox-sensitive CS derivative modified with Ch, sulfhydryl, and methoxy-PEG (mPEG) (mPEG-CS(SH)-CHO) was also investigated [55]. The optimized PTX-loaded micelles, prepared by an ultrasonication method, had an average micellar diameter of 158 nm, ZP of +26.9 mV, DLE of 11.7%, and DEE of 88.3%. The PTX-loaded micelles possessed excellent in vivo anticancer effect compared to both control group and Taxol formulation, as illustrated in Figure 7.

The PTX-loaded micelles exhibited a superior tumor inhibitory effect than Taxol, with a tumor inhibition rate of 67.3% vs. 53.8%.

To the best of our knowledge, no studies were conducted using oligochitosan for the synthesis of amphiphilic block copolymers, which are much easier to characterize than graft copolymers, for the further use as micellar DDS.

### 4.2. Drug Delivery Systems Based on Anionic Polysaccharides

**Figure 8:**
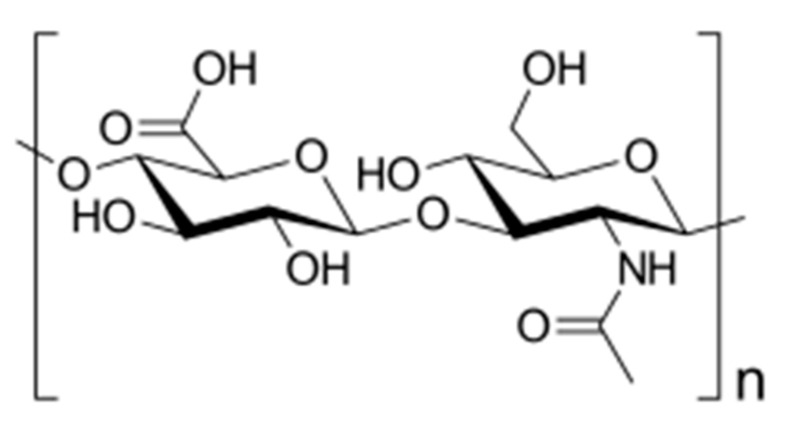
Chemical structure of hyaluronic acid (HA).

Hyaluronic acid or hyaluronate (HA), a natural linear anionic biopolymer composed of d-glucuronic acid and *N*-acetyl-d-glucosamine alternating units (Figure 8), is biodegradable, biocompatible, nontoxic, non-thrombogenic, non-immunogenic, and non-inflammatory. Owing to these extraordinary properties, as well as to its easy chemical modification, it was possible to prepare various HA-based biomaterials with promising biomedical applications [56].

HA–SS-deoxycholic acid amphiphilic copolymer micelles were prepared and used for the loading, by dialysis, and release of PTX, as an antitumoral model drug [57]. The increase of the DS from 3% to 10% led to a decrease of both the C.M.C. values, from 161.3 to 34.7 mg/L, and average micellar diameters, from 292.4 to 128.1 nm. On the contrary, ZP values increased from −31 to −36.6 mV. Maximum DLE and DEE values were 34.1% and 93.2%, respectively and the drug-loading step induced a decrease of the micellar sizes and an increase of the ZP values. In the absence of glutathione (GSH), a release of 14.3% within 24 h was noticed, whereas in the presence of 10 mM GSH, the cumulative release was 52.1% and increased up to 90% at a concentration of 20 mM GSH due to the redox sensitivity of disulfide bonds. In another study, PTX was loaded in micelles of hydrophobically modified HA with C6 or C18 acyl chains copolymer [58]. Compared to the previous study, lower maximum DLE (14%) and DEE (70%) values were calculated. The average diameters of the spherical PTX-loaded micelles, prepared by a solvent evaporation method, ranged from 57 to 66 nm. Very recently, PTX was also loaded in mPEG–SS–HA–C16 graft copolymers micelles [59]. The optimal characteristics of these spherical micelles, obtained by sonication, were 168.6 nm, −38.3 mV, and a C.M.C. value of 1.05 × 10^−2^ mg/mL. Moreover, the maximum values of DLE and DEE were 14.7% and 86%, respectively. The drug release was studied in the absence and in the presence of 10 mM GSH, and it appeared that in the absence of GSH, a slow drug release (10.8%) was observed within 24 h, whereas in the presence of GSH, the cumulative release reached 74.3% within 24 h.

Cholesterol (Ch) was another hydrophobic molecule used for the modification of HA [60]. The obtained micelles, with average diameters smaller than 100 nm were loaded with Dox, superparamagnetic iron oxide nanoparticles (SPION), and magnetic resonance imaging (MRI) contrast agents by a dialysis method. The release of Dox was studied in the absence and in the presence of hyaluramidase enzyme, which degrades the HA and enhances the drug release. Moreover, higher micellar uptake was observed in cancer cells versus normal cells. In another study, hydrophobic Ch was loaded in micelles based on hydrophobically modified HA with 1,2-dimiristoyl-sn-glycerol-3-phosphatidylethanolamine (DMPE) or 1,2-distearoyl-sn-glycerol-3-phosphatidylethanolamine (DSPE) [61]. HA-DMPE micelles were characterized by a C.M.C. value of 16.5 μg/mL, an average diameter of 165 nm, and a ZP value of −21.9 mV, whereas the HA-DSPE had a C.M.C. value of 13.4 μg/mL, an average size of 214 nm, and a ZP value of −29.1 mV. An increase of the average diameters as well as a decrease of the ZP values was noticed after the drug loading.

A series of HA–graft–octodecylamine graft copolymers, with DS values ranged from 12% to 51%, were synthesized for the preparation of Dox-loaded micelles [62]. Increasing the DS led to a decrease of both the average diameters, from 221 to 109 nm, and ZP values, from −23 to −10 mV. DEE and DLE, which were also affected by the DS variation, have maximum values of 95.3% and 16%, respectively. The copolymer micelles obtained at a DS of 12 had the highest release rate, whereas the slowest was observed for a DS of 51. However, in vitro cytotoxicity and cellular uptake studies showed that micelles with a DS of 23 are characterized by the highest anticancer activity and internalization. In a more recent study, dodecylamine was grafted to HA, at different DS, in order to prepare pH-sensitive graft copolymer micelles loaded with Dox by dialysis and ultrasonic methods [63]. Increasing the DS from 17.3 to 28.4, the C.M.C. values decreased from 38.0 to 21.4 μg/mL, as expected. The increase of the DS led also to a slight increase of the DEE, from 68.9% to 84.3%, whereas DLE values remained almost constant in the range of 25% to 27%. Moreover, the loading of Dox increased slightly the micellar size (around 160 nm) and decreased the ZP values. Furthermore, the release of Dox was investigated as a function of pH, and it appeared that the release rate was higher at pH = 5.0 compared to pH = 7.4. The authors showed that these micelles exhibited great antitumor efficiency with low in vivo systemic toxicity. A dual encapsulation of Dox and Cur was carried out in micelles based on HA, vitamin E succinate (VES) and PEG2000 (TPGS2K) derivatives [64]. HA-graft-TPGS2K micelles were characterized by smaller average micellar diameter (153 nm) and ZP value (−10.4 mV) than HA-graft-VES micelles, which had an average size of 223 nm and a ZP value of −10.4 mV. However, no significant difference was noticed between these two types of micelles concerning the DLE values (around 7%). The release of Dox and Cur was pH-dependent with a fast release at pH = 4.5 and pH = 5.5 but a slow rate at pH = 6.5 and pH = 7.4. Moreover, an improved in vivo antitumoral effect was observed, however, without a clear difference between the studied micellar systems.

Docetaxel (DTX) and SPION were co-encapsulated in hexadocylamine-modified HA micelles for dual tumor targeted MR imaging and combined chemo-phototherapy [65]. Micelles were obtained by dialysis, and the drug-loading step led to a decrease of both the average micellar diameters, from 150 to 130 nm, and ZP values from −28 to around −17 mV. It appeared that the DTX release could be accelerated by external near infra-red (NIR) laser irradiation in order to have an on-demand drug release at the tumor site. DTX was also loaded in micelles based on reduction responsive core-crosslinked HA-block-poly(trimethylene carbonate-co-dithiolene trimethylene carbonate) (HA-CCMs) block copolymer [66]. The micelles, obtained by a precipitation method followed by dialysis, loaded with 6.6 wt % DTX displayed a narrow size of 85 nm. A further increase of the drug amount up to 20% led to an average diameter of 94 nm. The ZP values were in the range of −26 to −29 mV. The maximum DLE and DEE values were 40% and 9.1%, respectively. The cumulative release of DTX after 24 h in the absence of GSH was around 20%, whereas it was almost 100% in the presence of 10 mM GSH, indicating the rapid reduction–responsivity of micelles. These micellar systems demonstrated a long blood circulation time, strong tumor accumulation, and deep tumor penetration.

**Figure 9:**
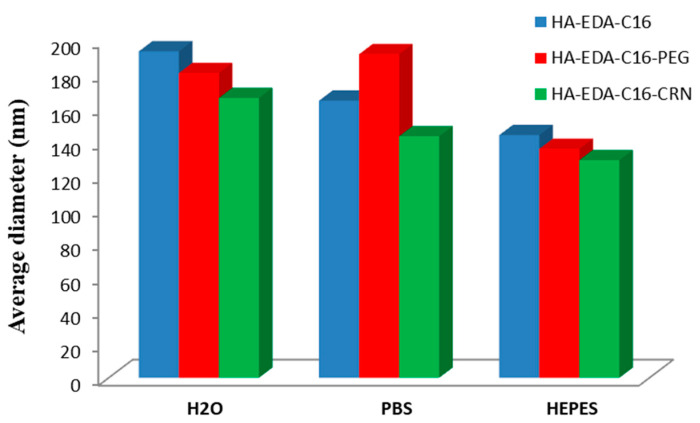
Variation of average micellar diameters of drug-loaded micelles in bidistilled water, PBS and 4-(2-hydroxyethyl)piperazine-1-ethanesulfonic acid (HEPES) buffer (redrawn from [67]).

Bongiovi et al. [67] have prepared an interesting micellar system, based also on HA, hexadecyl chains (C16), ethylenediamine (EDA), PEG, and/or l-carnitine (CRN), which was loaded with imatinib, a small molecule kinase inhibitor of ABL1. The maximum DLE value of 13% was obtained for HA–EDA–C16 micelles, whereas lower values were noticed for HA–EDA–C16–PEG and HA–EDA–C16–CRN micelles. The average diameters of both blank and drug-loaded micelles was influenced by the dispersion medium, smallest values being generally observed in 4-(2-hydroxyethyl)piperazine-1-ethanesulfonic acid (HEPES) medium. Figure 9 illustrates the variation of the average diameters of drug-loaded micelles as a function of the dispersion media.

The potential advantage of these micellar systems is that they can be administered non-invasively, i.e., by topical ocular instillation, and it appeared that these imatinib-loaded micelles were able to interact with corneal barrier and promote imatinib transcorneal permeation and penetration.

**Figure 10:**
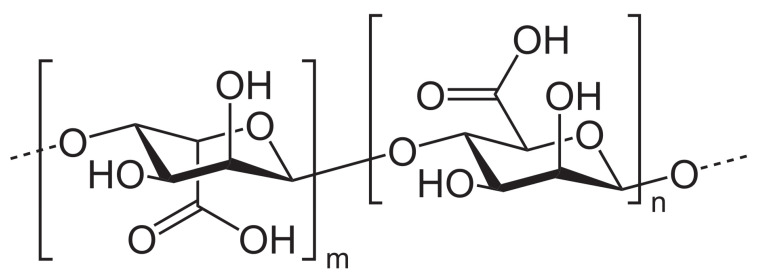
Chemical structure of alginic acid.

Alginate, another naturally occurring anionic polysaccharide, is composed of two conformational isomer residues: β-d-mannuronic acid (M) and α-l-guluronic acid (G), as illustrated in Figure 10.

The potential applications of alginate biopolymers are restricted because of its low water solubility and high solution viscosity; therefore, their chemical modification is necessary [68].

Poly(*N*-isopropylacrylamide) (PNIPAM) sequences were grafted on alginate backbone in order to obtain thermo-sensitive alginate–graft–PNIPAM copolymer micelles which were loaded with Dox by dialysis at 37 °C [69]. At this temperature, PNIPAM sequences become hydrophobic and form the micellar core. Molecular weight of PNIPAM grafts, DS, and copolymer concentration have an important influence of the colloidal characteristics of these micelles. For example, the increase of the DS from 5 to 20 has as a consequence the decrease of the C.M.C. values from 0.04 to 0.12 mg/mL, as expected. Moreover, increasing the M_n_ of the PNIPAM sequence, from 1000 to 4000 g/mol, led to an increase of the average diameters from 400 to around 1000 nm. Dox was loaded into these micelles with a maximum DLE value of 62.7% and a DLL of 24.2%. A sustained release of Dox, more than 90%, was achieved within 96 h. Yu et al. [70] have obtained similar thermo-sensitive sodium alginate–graft–PNIPAM graft copolymer micelles for the loading and delivery of 5-FU. However, in this case, the core of the micelles was constituted by sodium alginate crosslinked with metal ions (Ba^2+^, Zn^2+^ and Co^2+^) whereas the thermo-sensitive PNIPAM formed the corona. As a function of the metal ion used as crosslinking agents, the average diameters ranged from 50 to 340 nm. Moreover, the ZP varied from −22 to −27 mV. Furthermore, maximum DLE and DEE values were obtained for micelles crosslinked with Ba^2+^. Increasing the temperature and ionic strength or decreasing pH, a fast 5-FU release was observed.

Dodecyl glycidyl ether (DGE) was used for the hydrophobic modification of alginate in order to prepare drug-loaded micelles [71]. Sudan IV was encapsulated in these micelles, as a hydrophobic model drug. Owing to their amphiphilic behavior, the surface tension was investigated and values in the range of 25 to 30 mN/m were determined at a C.M.C. value of 0.1 mg/mL. The average diameters of these spherical micelles increased from 1 to 5 μm with increasing the copolymer concentration, whereas an almost constant ZP value of −82.85 mV was noticed independent of the copolymer concentration. Another hydrophobically modified and pH-responsive amphiphilic alginate copolymer was synthesized via Ugi reaction and used for the preparation of drug-loaded micelles by direct dissolution [72]. The impact of Na^+^ concentration on the micellar sizes was studied, and it appeared that the decrease of the ionic strength led to an increase of the average micellar diameters. TEM images showed that the mean micellar sizes were between 30 and 200 nm. The loading of a model pesticide (acetamiprid) was also influenced by the ionic strength and pH.

### 4.3. Drug Delivery Systems Based on Non-Ionic Polysaccharides

**Figure 11:**
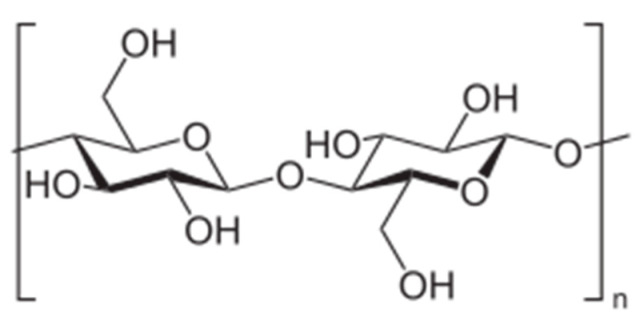
Chemical structure of cellulose.

Cellulose, composed of β-1,4-linked d-glucose units (Figure 11), is the most abundant biopolymer and has very interesting properties, such as biodegradability, renewability, high strength, and high modulus, but its practical applicability is limited by extremely poor solubility in water and common organic solvents. In order to be used as micellar DDS, cellulose has to be modified for the preparation of amphiphilic copolymers.

Amphiphilic cationic cellulose (HMQC) copolymers composed of long-chain alkyl groups, as hydrophobic sequences, and quaternary ammonium groups, as hydrophilic moieties, were used for the preparation of preduisone acetate-loaded micelles with average radii in the range of 320 to 430 nm [73]. The drug-loading step decreased the micellar sizes but increased slightly the ZP values, which ranged from 56.5 to 61.9 mV. The spherical morphology of these micelles, prepared by a dialysis method, was observed by TEM. Both DLE and DEE values were in the range of 16% to 22%, and a cumulative drug release of 86.5% was observed after 120 h, indicating a controlled and sustained drug release. In another study, cellulose was modified with 4-(*N*-methylamino) butyrate hydrochlorides for the preparation of camptothecin (CPT)-loaded micelles [74]. The micelles were prepared by a dialysis method, and their spherical morphology was assessed by SEM. As previously reported, the drug encapsulation process, with an encapsulation efficiency of about 86.4%, led to a decrease of the average micellar diameters from 495 to 233 nm due to stronger hydrophobic interactions between the drug and the hydrophobic micellar core. The drug release tests indicated a sustained release of CPT to >80% over 4 days at both pH = 6.8 and pH = 7.4.

Amphiphilic cellulose–graft–poly(p-dioxanone) (MCCC-graft-PPDO) copolymer micelles were prepared by the dialysis method in order to encapsulate hydrophobic fluorescent conjugated polymers (FCPs) for tumor cell imaging [75]. After the loading of FCPs, the average micellar diameters increased from 40 nm to about 100 ÷ 200 nm and were stable over time. Moreover, DLL values increased with increasing the FCPs concentration, whereas the DEE values decreased. Furthermore, it was demonstrated that the optimal DEE and DLL values could by obtained with 1000 μg/mL MCCC–graft–PPDO and 75 μg/mL FCPs, and that these FCPs-loaded micelles are successfully uptaken by the cancer cells and located at the cytoplasm of the cells, suggesting their great potential for tumor cell imaging and early detection of tumor cells.

Carboxymethyl cellulose–graft–polylactic acid (C.M.C-graft-PLA) copolymer was functionalized with anti-EpCAM antibody by an amidation reaction for the active delivery of both Dox and HepG2 cells [76]. These drug-loaded micelles, prepared by an emulsification method followed by dialysis, have average micellar diameters of around 150 nm and were stable for 7 days. Moreover, these micelles were suitable for intravenous injections, as the C.M.C. was low (0.043 mg/mL). Moreover, by increasing the amount of loaded drug, it appeared that DLL increased from 8.2% to 12.6%, whereas DEE decreases from 81.6% to 36%. As the pH value decreases, the solubility of Dox increases, and therefore, the drug release rate from these micelles will increase. Furthermore, the functionalized Dox-loaded micelles exhibited better antitumor and therapeutic effects than both free drug and non-functionalized drug-loaded micelles.

**Figure 12:**
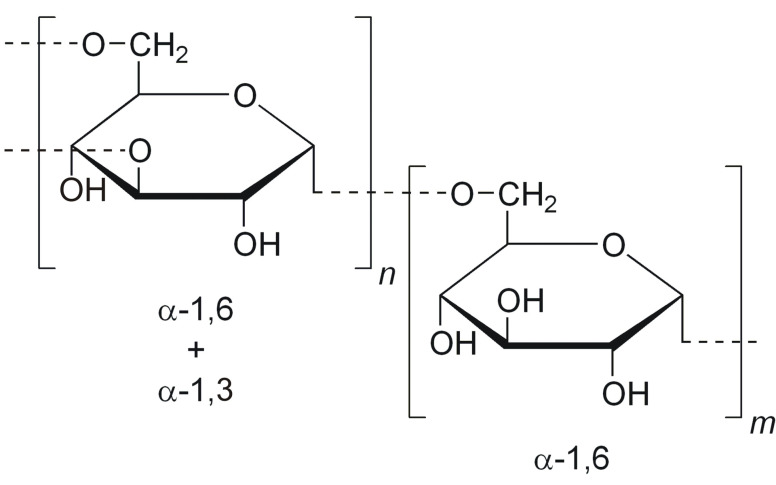
Chemical structure of dextran (DEX).

Dextran (DEX), composed predominantly of α-1,6-linked glucopyranose units with a low degree of 1,3-branching (Figure 12), is a highly water-soluble polysaccharide of bacterial origin, produced by *Lactobacillus*, *Leuconostoc*, and *Streptococcus* species. In order to prepare amphiphilic copolymers, DEX was hydrophobically modified with different synthetic polymers or other hydrophobic molecules.

An interesting study was related to the synthesis of an amphiphilic DEX–SS–poly(ε-caprolactone) (PCL) block copolymer in which PCL was linked to DEX by a disulfide bond [77]. Dox was loaded, by a dialysis method, into micelles having a C.M.C. value of 9.3 mg/L. The drug-loading step increased the micellar average diameter from 60 to 80 nm. The DLE value was around 70%, and a minimal drug release (<20%) was noticed within 20 h. From the in vitro tests, it appeared that Dox loaded in these reduction–responsive biodegradable micelles is delivered and released to the cytoplasm as well as to the cell nucleus, achieving higher drug efficacy, due to the reduction–responsive SS bonds, than the drug loaded in reduction-insensitive Dex–PCL micelles. More recently, Dox was also loaded, by dialysis, into DEX–graft–PCL graft copolymer micelles [78]. The maximum values for DEE (42.2%) and DLE (17.4%) were obtained for a Dox concentration of 0.3 mg/mL. The cumulative release at pH = 5.2 was greater than that at pH = 7.4, which was probably due to the protonation of Dox in acidic environment. Furthermore, the cytotoxicity tests reflected the biocompatibility of the micelles. In another study, Dox was also loaded in DEX–graft–stearate amphiphilic graft copolymers micelles [79]. Depending on both the DS of SA and the molar mass (M_n_) of DEX, the C.M.C. values ranged from 0.01 mg/mL to 0.08 mg/mL whereas the hydrodynamic diameter ranged from 24 to 258 nm. However, in this study, the drug loading has not affected the micellar size. DEE values reached up to 92.23%, while a maximum DLL value of 14.41% was determined. A prolonged in vitro Dox release, up to 48 h, was noticed as a function of the M_n_ of DEX, DS of SA, or the drug-loading content. Moreover, in vivo antitumor activity tests showed that the tumor growth was suppressed to a higher extent in the presence of these Dox-loaded micelles as compared with commercial Dox injection. Another group has prepared the same type of graft copolymer by the acylation of DEX with stearoyl chloride, at different DS, but in this study, etoposide was used as a model drug [80]. Compared to the previous study, an increase of the average diameters of these micelles was observed after the drug loading, whereas an opposite effect was noticed for the ZP values. For example, for the DEX_6_-SA_7.5_ sample, the average diameter increased from 131.7 to 169.6 nm, but the ZP decreased from −30 to −19.2 mV. Moreover, it appeared clearly that the DS has a direct influence on both the DEE and DLE, the maximum values being 31.7% and 93.3%, respectively. A maximum cumulative drug release of around 80% was observed after 48 h in PBS at pH = 7.4. In another study, DEX was further modified with stearyl acid, and the obtained micelles, with a C.M.C. value of 1.8 mg/L, were loaded by a precipitation method with both Dox and supermagnetic iron oxide (SPIO) nanocrystals [81]. These drug-loaded micelles were characterized by an average diameter of 100 nm and showed good biocompatibility and excellent internationalization ability against MCF-7/Adr cells. Dox and SPIO nanoparticles were also encapsulated in A54 peptide-functionalized DEX–graft–PLGA graft copolymer micelles with a C.M.C. value of 22.51 μg/mL [82]. The average micellar diameter increased from 50 to 100 nm after the drug loading with DEE and DLE values of 80% and 4%, respectively. A cumulative Dox release of around 80% was noticed after 72 h in PBS at both pH = 5.5 and pH = 7.2. Moreover, these peptide-functionalized Dox-loaded micelles showed better therapeutic effects than the non-functionalized micelles. Dox was also loaded, via a precipitation method, into DEX–block–PLA block copolymer micelles with average diameters in the range of 15 to 70 nm, as a function of the molecular characteristics of the copolymer [83]. Maximum DLE and DEE values of around 21.2% and 45%, respectively were determined. In vitro hemolysis results showed that these micellar systems are suitable as injectable DDS. More recently, other Dox-loaded DEX–block–PLA micelles, with average diameters ranged from 158 to 182 nm, were prepared by a dialysis method [84]. The C.M.C. values decreased from 6.63% to 3.87% as the M_n_ of the PLA sequence increased from 2000 to 4000 g/mol. Moreover, the maximum DLE and DEE values were 12.6% and 75%, respectively. A maximum cumulative drug release of around 60% was observed after 72 h in PBS at 37 °C and pH = 7.4. In a similar study, Dox was loaded into DL-lactide-co-glycolide-block-DEX spherical micelles by a dialysis method [85]. After the drug loading, the average micellar diameters increased from 82.6 to 206 nm for a drug content of 12.3%. In addition, it appeared that higher initial drug feeding induced higher drug content and slower release rate.

**Figure 13:**
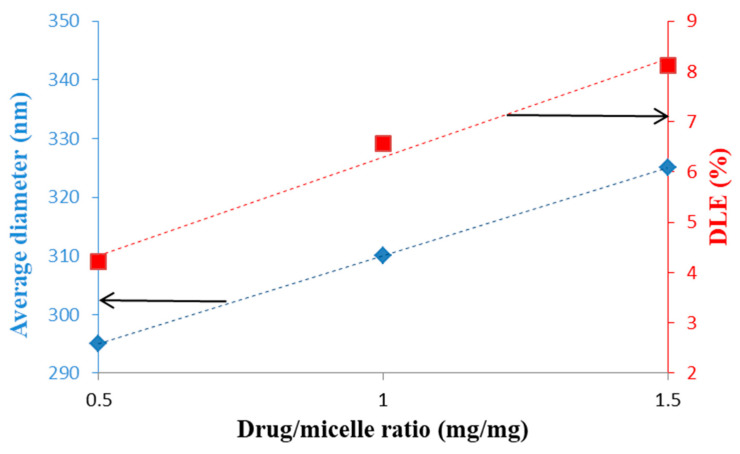
Evolution of the average micellar diameters and DLE as a function of the Dox/micelle ratio (mg/mg) (redrawn from [86]).

The same model drug, Dox, was loaded, by Yu et al. [86], with DEX-graft-α-tocopherol succinate graft copolymer micelles prepared by a dialysis method. Both micellar sizes and DLE were increased with increasing the drug/micelles ratio and this tendency is illustrated in Figure 13.

On the contrary, this increase of the Dox/micelle ratio has as consequence the decrease of DEE values from 87.9% to 58.9%. A spherical morphology was observed by TEM and a release percentage of 50.2% was determined after 96 h. This slow release was induced either by the hydrophobic/hydrophobic or van der Waals interactions between Dox and the hydrophobic sequences of the copolymer. In vitro cytotoxicity studies revealed that these drug-loaded micelles were practically non-toxic and therefore suitable as DDS.

Another type of DEX-based DDS was related to Cur-loaded DEX-graft-casein graft copolymers micelles with an average diameter of 75.3 nm and a ZP value of −18.1 mV [87]. The stability of these micelles was determined by turbidimetry in the pH range of 2 to 8, and it appeared that these PMs are more stable than the casein micelles. Another group has loaded Cur, by dialysis, in oxidized DEX–SA spherical micelles with average diameters smaller than 50 nm [88]. The C.M.C. values ranged from 0.07 to 0.158 mg/mL as a function of the DS of SA. As expected, the DLE values increased with increasing the initial drug feeding, whereas an opposite effect was noticed for DEE. Moreover, in vitro drug release was prolonged by adjusting the amount of loaded Cur. Cur was also loaded in other amphiphilic block copolymer micelles based on two types of DEX, such as hydrophilic and hydrophobic acetalated DEX. This double-based DEX copolymer self-assembles in water at a low C.M.C. value of 12 mg/L. The Cur loading, by dialysis, led to a slight decrease of the average micellar diameter from 103 to 98.9 nm. In addition, the drug loading decreased the ZP values from 2.8 to −6.1 mV. The degradability of this micellar system under acidic conditions was investigated, and it appeared that demicellization occurs after 2 h at pH = 5.5 due to the cleavage of acetal groups. On the contrary, it was shown that this micellar system is stable up to four months at a neutral pH value.

Blanco-Fernandez et al. [89] studied a thermo-sensitive micellar system based on DEX grafted with poly(isopropylacrylamide) (PNIPAM) and loaded with methotrexate (MTX) at two pH values, such as 5.5 and 7.4. Only unimers were observed at 20 °C, whereas at 37 °C, the C.M.C. value was 80 μg/mL. At 37 °C and a copolymer concentration of 1 mg/mL, the average micellar diameter was 190 nm. Moreover, the drug-loading efficiency was higher at pH = 7.4 than at pH = 5.5. As expected, MTX-loaded micelles showed pH- and thermo-responsive release and a higher cytotoxic effect than that of free MTX. Another pH-sensitive graft copolymer with controlled M_n_ and narrow dispersity, based on DEX and poly(oleic acid), has been synthesized by reversible addition-fragmentation chain transfer (RAFT) polymerization [90]. By dialysis, were obtained spherical micelles, from a C.M.C. value of 0.04 mg/mL, which were further loaded with nifedipine (NFD), as a model drug. Increasing the copolymer concentration from 0.03 to 0.05 mg/mL led to the increase of the average micellar diameters from 201.5 to 264.3 nm. Moreover, the drug loading increased slightly these diameters and the ZP value from −16.7 to −28 mV. Furthermore, DEE and DLE values were calculated to be 41.42% and 10.35%, respectively. The NFD release rate was faster at pH = 1.2 than at pH = 7.4.

Starch is composed of amylose, a linear polymer of glucose unit with α-(1→4) linkage, and amylopectin, which is highly branched with lots of short chains that linked through α-(1→6) linkage. In order to overcome the drawbacks of native starch, such as poor processability and solubility in common organic solvents, retrogradation and syneresis, low shear stress resistance and thermal decomposition, several types of modifications are possible: physical, chemical, enzymatic and genetic engineering, respectively [91].

The core of starch–graft–PEG2000 or 5000 micelles was crosslinked in the presence of lipoic acid via disulfide bonds in order to study the reduction–responsive behavior in the presence of GSH [92]. Dox was loaded, by a dialysis method, as a model drug, and the crosslinking of the micellar core increases both DLE and DEE values, the maximum values being equal to 9.52% and 57.12%, respectively. Moreover, this core crosslinking led to a decrease of the average micellar diameters from around 300 to approximatively 200 nm. In the presence of 10 mM GSH, these micelles exhibit faster drug release behavior and higher cellular proliferation inhibition efficiency. In another similar study, 3,3′-dithiodipropionic acid (DPA) was used as crosslinking agent in order to obtain Dox-loaded micelles, by a direct dissolution method, with a hydrophobic crosslinked starch core and a hydrophilic mPEG corona [93]. The crosslinking process induces the decrease of both average micellar diameters and C.M.C. values due to the formation of a more compact hydrophobic core. The micellar stability was investigated during 48 h in PBS at 37 °C, and it appeared that only a slight increase of the micellar sizes occurred during this period. However, an important increase of the micellar size was observed, due to the cleavage of the SS bonds and thus micellar aggregation, in 10 mM GSH solution. As previously indicated, this reduction–responsive behavior has an influence also on the Dox release rate.

Another amphiphilic glucose-sensitive dialdehyde starch polymer containing 3-aminophenylboronic acid (APBA) as a glucose-responsive group and mPEGylated dialdehyde starch (mPEG-DAS) with hydrophobic 7-hydroxycoumarin-4-acetic acid (Cou) was synthesized by Wen et al. and used for the preparation of insulin-loaded micelles by a sonication method [94]. The conjugation of Cou decreased the micellar size from 241.4 to 177.9 nm, and the C.M.C. values increased as the DS of Cou increased. Insulin was loaded into these micelles with a DLE of 30.4% and a DLL of 9.4%. A cumulative drug release of around 55% was observed within 50 h in the presence of 3 g/L glucose while demonstrating comparatively inert release at a glucose concentration of 1 mg/mL. A similar study was conducted by the same research team [95]. However, in this case, sulfobetaine formed the hydrophilic corona of the micelles.

Classical starch–graft–PEG graft copolymers were synthesized from tapioca starch biopolymer for the preparation of Cur-loaded micelles [96]. Empty spherical micelles, prepared by direct dissolution, had an average micellar diameter around 100 nm and a C.M.C. value of 0.0765 mg/mL, at pH = 7.4. A maximum DLE value of 5.6% and a DEE of 39.4% were obtained when 100 mg of polymer was added to 10 mg of Cur. After 95 h, cumulative Cur release has higher at pH = 5.5 (23%) than at pH = 7.4 (8%). In a similar study, PEG 5000 and TG100-115, which is an exclusive PI3Kc inhibitor, were conjugated on hydroxyethyl starch in order to obtain sorafenid-loaded micelles by a dialysis method [97]. The drug release was investigated as a function of pH (5.5, 6.8, and 7.4), and it appeared that the higher release rate was observed at low pH value. Moreover, a pharmacokinetic study showed that these micelles had longer half-life than the solution of the free drug, which was favorable for high propensity of extravasation through tumor vascular fenestrations. Furthermore, under low pH and high a-amylase reductive conditions, a quick demicellization occurs, thus enhancing significantly the cytotoxic activity against Hep-3B liver cancer cells. Finally, starch was modified with octenyl succinic anhydride in order to obtain β-carotene-loaded micelles [98]. As expected, the C.M.C. values depended on both the DS and M_w_ of the graft copolymer. Empty spherical micelles, with sizes smaller than 20 nm, were prepared by direct dissolution in hot water and the drug loading led to an important increase of the average micellar diameters up to 1000 nm.

### 4.4. Drug Delivery Systems Based on Miscellaneous Polysaccharides

Table 3 gives several examples of drug delivery systems based on less studied polysaccharides.

From Table 3, it appears that dialysis is generally the preferred method for the preparation of biopolymers-based micellar DDS.

## 5. Micellar Drug Delivery Systems Based on Peptides

A high number of plant and animal sources have been used for the isolation or production of peptides [117]. Peptides exhibit good biological functions and are well tolerated upon administration, as they are derived from natural sources. In addition, they bind specifically to their receptors, thus avoiding non-specific interaction with other parts of the body, thereby preventing unwanted adverse effects. Peptides are often modified chemically to make them less prone to degradation by proteolytic enzymes and therefore more stable in vivo. PEGylation provides steric stabilization that prevents recognition by opsonins and decreases cleavage by proteases, thus improving the half-life of peptides in vivo. PEGylation also provides stability to peptide molecules against aggregation [118].

The active tumor targeting and tumoral pH-responsive polymeric micelles were prepared by mixing AP peptide (CRKRLDRN) conjugated PEG–poly(d,l-lactic acid) block copolymer (AP–PEG–PLA) into the pH-responsive micelles of methyl ether poly(ethylene glycol) (MPEG)–poly(β-amino ester) (PAE) block copolymer (MPEG-PAE) [119]. These mixed amphiphilic block copolymers were self-assembled, by a thin-film hydration method, to form stable AP peptide-conjugated and pH-responsive APPEG–PLA/MPEG–PAE micelles (AP-pH-PMs) with an average size of 150 nm. Dox, as a model anticancer drug, was efficiently encapsulated into the AP–pH–PMs (Dox–AP–pH–PMs) with loading efficiencies higher than 82%. The in vivo antitumor efficacy Dox–AP–pH–PMs was evaluated in MDA-MB-231 human breast tumor-bearing mice, and it appeared that Dox–AP–pH–PMs-treated mice showed significant antitumor therapeutic efficacy, indicating that AP–pH–PMs could successfully deliver the encapsulated Dox at the target tumor.

Peptide–PEG conjugates self-assembled into spherical micelles, so-called 3-helix micelles, with an average diameter of 15 nm [120]. Dox was used as a model drug in order to estimate the drug-loading capacity (around 8 wt %), and the drug loading was performed using a dry-down method. By DLS, it appeared that the drug encapsulation has not affected the micellar sizes. In vivo pharmacokinetics and biodistribution tests clearly demonstrated that these 3-helix micelles achieved long circulation half-life and efficient clearance. Moreover, once the peptide is enzymatically degraded, the micelle will disassemble, releasing the loaded cargo. A further advantage is represented by the fact that all the biocompatible components of the micelles will be metabolized.

R3V6 peptides, which were composed of three arginines and six valines, self-assembled into micelles, and the core of these micelles was further loaded with dexamethasone, by an oil-in-water emulsion/solvent evaporation method, in order to improve their transfection efficiency [121]. It was demonstrated that the R3V6–dexamethasone had higher anti-inflammatory effect than dexamethasone alone. R3V6 amphiphilic peptide micelles were also loaded with bis-chloroethylnitrosourea (BCNU) for efficient delivery into brain tumors [122]. In vivo evaluation revealed that R3V6 efficiently delivered BCNU into the cancer cells in an intracranial glioblastoma animal model, indicating that R3V6–BCNU are more effective in reducing the tumor size than BCNU alone. In addition, R3V6 did not induce cytotoxicity to the cells. Other R3V6 peptide micelles were prepared and evaluated as a Cur carrier to INS-1 insulinoma cells [123]. The drug encapsulation was carried out using an oil-in-water emulsion/solvent evaporation method. The size and surface charge of the Cur-loaded micelles were approximately 250 nm and 17.49 mV, respectively. These micelles, with Cur being in the core of the R3V6 micelles, delivered Cur to the INS-1 cells more efficiently than either free Cur or a simple blend of R3V6 and curcumin. Therefore, it was demonstrated that R3V6 peptide micelles are effective nanocarriers, and that Cur-loaded micelles may improve the viability of pancreatic b-cells in islet transplantation.

The peptide amphiphile micelles (PAMs) boost peptide-specific immune responses without causing any undesirable side effects. In the study of Accardo et al. [124], PAMs were prepared and used as potential synthetic self-adjuvant vaccines to treat Herpes simplex virus (HSV) infection. Pure and mixed micelles, based on epitopes gB_498−505_ (H-L-Ser-Ser-Ile-Glu-Phe-Ala-Arg-Leu-Amide) and gD_301−309_ (H-L-Ser-Ala-Leu-Leu-Glu-Asp-Pro-Val-Gly-Amide), which were selected from HSV envelope glycoprotein B (gB) and glycoprotein D (gD), were prepared by solid phase methods. Moreover, these peptides had their N-terminus modified with hydrophobic moieties containing two C18 hydrocarbon chains. Colloidal characterization of PAMs confirmed that they were sufficiently stable and also suitable for in vivo applications: critical micelle concentration values were around 4.0 × 10^−7^ mol/kg; hydrodynamic radii (RH) ranged between 50 and 80 nm, and all micelles had ZP values around −40 mV. The obtained experimental data indicated that these PAMs can exert significant immune modulatory effects on macrophage activation, leading to the production of predominantly IL-23 and other pro-inflammatory cytokines.

**Figure 14:**
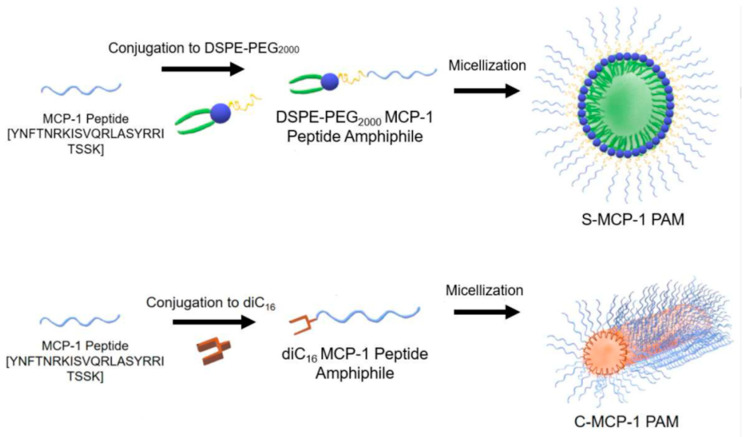
The monocyte chemoattractant protein-1 (MCP-1) peptide was conjugated to 1,2-distearoyl-sn-glycerol-3-phosphatidylethanolamine (DSPE)–poly(ethylene glycol) (PEG2000) to form spherical MCP-1 peptide amphiphile micelles (PAMs), or to diC16 tail to form cylindrical MCP-1 PAMs [126].

Monocyte chemoattractant protein-1 (MCP-1) is part of the family of C−C chemokines that promote monocyte and macrophage migration to sites of inflammation [125]. These authors synthetized MCP-1 peptide sequence containing the CCR2-binding motif (residues 13–35) and an additional N-terminal cysteine. Moreover, this peptide was conjugated to the DSPE–PEG2000 lipid tail, via a thioether bond, in order to obtain PAMs through a thin-film hydration method. TEM analysis confirmed the spherical morphology of these micelles, while by DLS, an average hydrodynamic diameter of 13.6 ± 2.4 nm was determined. In addition, MCP-1 PAMs displayed a slightly positive zeta potential of 11.0 ± 0.1 mV. This study demonstrated that the binding affinity and chemoattractant properties of MCP-1 PAMs were enhanced compared to the free peptide and that MCP-1 PAMs reduced the proliferation of prostate cancer cells by decreased CCR2 expression. Both cylindrical and spherical PAMs, based on monocyte chemoattractant protein-1 (MCP-1), were also obtained by Joo et al. [126] by a dry film hydration method. The schematic representation of these micelles is given in Figure 14.

Transmission electron microscopy (TEM) confirmed the spherical and cylindrical morphology of micelles with diameters of 14.9 ± 6.0 nm for S-MCP-1 PAMs. Similarly, the diameters of C-MCP-1 PAMs were measured to be 10.7 ± 0.3 nm with lengths of 62.2 ± 41.3 nm. As expected, slightly higher values were determined by DLS. S-MCP-1 had a zeta potential value of 1.5 ± 0.6 mV, which was smaller than that of C-MCP-1 (17.6 ± 5.7 mV). The diameters of both PAMs in Dulbecco’s modified eagle medium (DMEM) supplemented with 10% fetal bovine serum (FBS) steadily increased over 12 h, suggesting that FBS adsorbed onto the micellar surface. On the contrary, both PAMs in PBS exhibited no significant changes in particle size. Furthermore, it appeared that both type of micelles were biocompatible with monocytes and enhanced the secondary structure of the MCP-1 peptide, thereby improving the ability of the micelles to mimic the native MCP-1 protein structure. However, cylindrical PAMs showed a greater ability to attract monocytes compared to spherical PAMs.

In order to provide an active targeting delivery, PAMs were functionalized onto the surface with aptamers, which are ADN oligonucleotide sequences [127]. These micelles were prepared by a thin-film hydration method using a model peptide (RPDRKLEVFEKEFLRMELGER). The size of the aptamer-functionalized PAM was around 410 nm, by DLS, whereas by SEM, a much smaller size (110 nm) was observed. Moreover, ZP value was around −28 mV. It appeared also that these micelles are stable for over 4 h in model systems mimicking cellular and tissue environments. Furthermore, it was demonstrated that the aptamer enhanced the delivery to a specifically-targeted B-cell leukemia cell line.

Cyclic-Arg-Gly-Asp (cRGD) peptide was conjugated to acetal-poly(ethylene glycol)-b-poly(β-benzyl L-aspartate) (Ac-PEG-b-PBLA) copolymer and mixed with MeO-PEG-PBLA-Ac (1:3) in order to prepare epirubicin-loaded cRGD-installed micelles (cRGD-Epi/m) [128]. As control micelle (Epi/m), only MeO-PEG-PBLA-Ac polymer was used. Epirubicin, a potent antiglioblastoma agent, was loaded by a dialysis method through a pH-sensitive hydrazone-bond for the effective treatment of glioblastoma multiforme (GBM). A 12% drug loading efficacy was calculated, and the average diameter of these drug-loaded micelles was around 30 nm. The obtained results demonstrated significant antitumor effect of cRGD-Epi/m against orthotopic GBM, i.e., a 12-fold higher antitumor activity was displayed by cRGD-Epi/m than that of Epi/m.

Block co-polypeptides, based on γ-benzyl-l-glutamic acid, ε-*N*-benzyloxycarbonyl-l-lysine (ZK_30_-b-BnE_30_ and ZK_30_-b-BnE_60_), were prepared by a dialysis method [129]. Bedaquiline (BQ) was used as a model drug for the treatment of multidrug resistant tuberculosis. Moreover, sodium alginate was used for the coating of these micelles in order to increase their stability. The average diameter of the BQ-loaded polypeptide micelles was around 47.5 nm, whereas the coating with 20 wt % alginate led to an increase of this value to 435.8 nm. On the contrary, quite similar ZP values of −23.6 and −25.2 mV were determined. Moreover, the alginate coating led only to a slight decrease of the drug loading efficacy from 19.2% to 17.3%. Furthermore, alginate-coated micelles showed a 15-fold decrease of released BQ compared non-coated micelles and a sustained release in a complex medium such as human plasma. The release of BQ from alginate-coated micelles was also reduced in gastric and intestinal simulated media to values well below 25%. Finally, these BQ-loaded micelles showed a lower minimum inhibitory concentration compared to free BQ.

Another class of peptides is represented by the antimicrobial peptides (AMPs), which have played critical roles in the clinical therapy of drug-resistant bacterial infections. Zhang et al. [130] developed a novel AMP amphiphilic conjugate, DP7-C, by modifying a formerly identified highly active AMP with cholesterol. The average particle size of the prepared DP7-C spherical micelles was 36.06 ± 1.5 nm, with a polydispersity index (PDI) of 0.176 and a ZP value of 43.8 ± 0.27 mV. Compared to unconjugated counterparts, the DP7-C micelles showed lower hemolytic activity toward human red blood cells. Moreover, by eliciting specific immunomodulatory activities in immune cells, the DP7-C micelles exerted distinct therapeutic effects in zebrafish and mouse infectious models.

## 6. Micellar Drug Delivery Systems Based on Proteins

Proteins have emerged recently since they exhibit unique functions and properties and can be used as base materials for the production of DDS. Protein-based DDS have several advantages, such as biodegradability, stability, surface modification of particles, and ease of particle size control. Hong et al. [131] have reviewed the general aspects concerning the preparation and characterization of different types of NPs-based on proteins—however, without a detailed discussion of the micellar DDS based on proteins. Therefore, in this paragraph, we review the literature data concerning the protein-based DDS from the last decade. Table 4 lists the physicochemical properties of some of the protein polymers used for drug delivery.

Albumin can be obtained from different sources, such as egg white, bovine serum albumin (BSA), and human serum albumin (HSA). Albumin maintains the osmotic pressure in human blood and is responsible for binding of nutrients. Albumin is highly soluble at physiological pH and is an attractive carrier due to its ability to bind to various molecules but also to form conjugates with different polymers [132,133].

Several interesting studies were carried out by the team of Stenzel on the preparation of BSA-based drug delivery systems for cancer therapy [134,135,136,137,138]. Among all these reports, it is of interest to cite the study in which BSA was modified with PCL, and the obtained micelles were loaded with albendazole with a DLE of 16.4% [136]. These drug-loaded micelles, with sizes around 100 nm, exhibited important cytotoxicity and high inhibition effects on the MCTS growth, indicating that the system can be used for the treatment of pancreatic cancer. In another study, Cur-loaded drug delivery systems (DLE around 40%) were prepared by the co-assembly of BSA-PCL copolymer with poly(oligo(ethylene glycol) methyl ether acrylate)-poly(ε-caprolactone) (POEGMEA-PCL) copolymer [137]. A series of various hybrid nanoparticles was obtained with sizes in the range of 100 nm to 120 nm. Moreover, a decrease of the ZP value was noticed when the amount of BSA increased. Furthermore, higher amounts of BSA led to high cellular uptake values, and it appeared that can enhance the selectivity of albumin toward cancer cells.

A novel type of denatured albumin copolymer, based on polycationic bovine serum albumin precursor protein (cBSA-147) and PEG5000, was prepared in order to facilitate the encapsulation of Dox [139]. Spherical drug-loaded micelles with an average size of around 30 nm were obtained by a dialysis method. Moreover, these micelles had a highly positive surface charge due to the cationic nature of the cBSA-147 precursor protein. The encapsulation efficiency ranged from 13.1% to 51.6% as a function of the Dox/copolymer ratio. Cell imaging via confocal microscopy revealed fast cellular uptake of these micelles after only 1 h of incubation. Moreover, Dox uptake after encapsulation into micelles was fivefold increase compared to free Dox, indicating efficient intracellular drug release.

In other studies, human and bovine serum albumin were used, as targeting ligands, for the functionalization of polymer conjugate nanocarriers [140,141,142,143].

Casein represents about 80% of bovine milk protein and precipitates at pH = 4.6. Casein exists in fresh milk in the form of a micelle structure, which is a complex aggregate of proteins (α-, β-, and κ-casein) and colloidal phosphate calcium. The most common form of casein is sodium caseinate [144].

**Figure 15:**
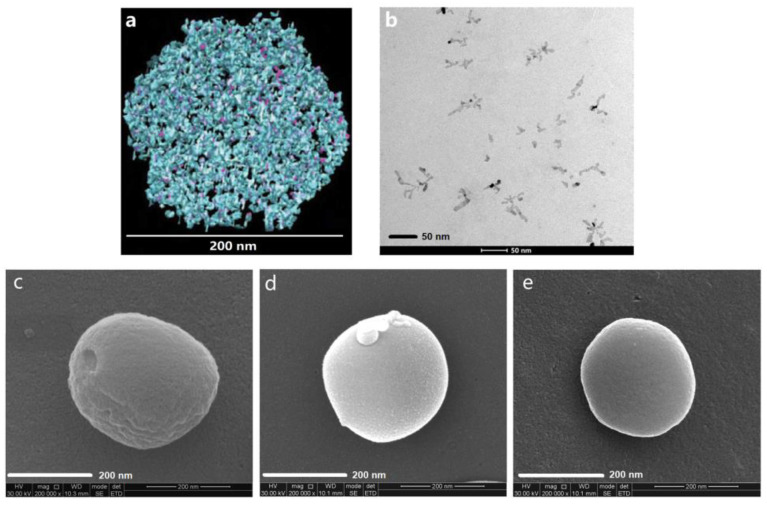
Modification of the casein-nanoparticles through phosphatidylcholine. (**a**) The image of the casein-nanoparticle derived from cryo-transmission electron tomography comes from the literature [151], with permission from Elsevier, 2011; (**b**) TEM image of phosphatidylcholine (PC); (**c**) SEM image of casein-nanoparticles; (**d**) SEM image of PC–casein nanoparticles, (**e**) SEM image of Cx-PC–casein NPs.

β-casein (β-CN) is an amphiphilic protein that self-organizes into well-defined core–shell micelles and therefore it has been used for the encapsulation of hydrophobic drug, such as mitoxantrone (MX) [145]. The drug was entrapped by stirring its dimethyl sulfoxide (DMSO) solution into PBS containing β-CN. However, the concentration of DMSO in PBS has not exceeded 6%. The optimal loading molar ratio was 3.3 MX/β-CN at 1 mg/mL β-CN, and a bimodal particle size distribution was noticed by DLS. The same research group has also studied PTX-loaded β-CN micellar systems [146]. β-CN micelles were also used for the encapsulation of celecoxib (Cx), as a model anti-inflammatory hydrophobic drug utilized for treatment of rheumatoid arthritis and osteoarthritis but also evaluated as a potent anticancer drug [147]. The empty micelles’ diameter measured by DLS was 19 ± 3 nm. After loading the micelles with 1:8 mole ratio of protein/drug, the diameter, as measured by DLS, increased to 31 ± 5 nm. The lyophilization process has not affected the micellar sizes. Empty β-casein micelles are highly negatively charged, having a ZP value of −30 mV. Moreover, the drug encapsulation led to an increase of the ZP up to −42 mV. High encapsulation of Cx in the Cx/β-CN formulation was achieved [148]. Incubating the Cx/β-CN micelles in aqueous buffer at 37 °C showed almost complete drug release after 30 min. In another study, the same research team showed that Cx/β-CN micelles are metastable supramolecular assemblies that transform upon heating to thermodynamically stable drug free β-CN micelles while releasing their drug load [149]. More recently, Xv et al. [150] showed that egg yolk phosphatidylcholine PC98T (PC) significantly increased the physicochemical stability of Cx/PC-β-CN NPs (192.6 nm) from 5 min (Cx/β-CN micelles) to 2.5 h at 37 °C. The images of these NPs are provided in Figure 15.

β-CN micelles were further used in order to orally deliver a synergistic combination of a chemotherapeutic drug (Paclitaxel) (PTX) and a P-glycoprotein-specific transport inhibitor (Tariquidar) (TQD), which were individually encapsulated within these micelles, for overcoming multidrug resistance (MDR) in gastric cancer [152]. Empty β-CN micelles in PBS showed a ZP value of −45.6 mV, and this value was not significantly affected by the encapsulation of the two drugs. A multimodal size distribution, depending on the drug/β-CN molar ratio, was noticed for both drug-loaded micellar systems. Moreover, the drug/β-CN molar ratio has an important influence on both loading and encapsulation efficacies. In vitro cytotoxicity results demonstrated that TQD achieved a complete MDR reversal in the human MDR gastric carcinoma cell line EPG85-257RDB. The same research group has showed in another study the modularity and versatility of this β-CN-based delivery system using a different synergistic drug duo to treat MDR gastric cancer cells overexpressing the breast cancer resistance protein (BCRP) [153]. The chemotherapeutic drug (SN-38), a BCRP transport substrate, and the BCRP efflux transport inhibitor, elacridar, were individually encapsulated within β-CN micelles, and similar conclusions were reached.

Naringenin, a hydrophobic flavanone with several beneficial biological effects, such as antioxidant, anticancer, and anti-inflammatory activity, has been loaded also in β-CN micelles [154]. As previously reported, the size distribution of the β-CN micelles was multimodal and depended of the drug/β-CN molar ratio. The ZP values change from about −22 mV at pH = 7 and 6 mV to 22 mV at pH = 2. Moreover, it was found that in the pH range of 3 to 5, the system is not stable, presumably because of the low net charge of the β-CN molecules. Furthermore, the addition of 1 mol/L NaCl led to the aggregation phenomena. By fluorescence spectroscopy, it appeared that naringenin binds with β-CN at neutral (pH = 7) as well as under acid conditions (pH = 2), and it was found that naringenin-containing β-CN micelles are more stable, having a lower C.M.C. value and a larger aggregation number (*N*agg) compared to pure β-CN micelles. Finally, the presence of naringenin had a clear influence on the structure of β-CN micelles, which were changed from an elliptic to a spherical morphology.

Sodium caseinate was recently used for the encapsulation of two hydrophilic nutraceuticals, namely epigallocatechin gallate (EGCG) and FA [155]. The encapsulation efficiency of EGCG was 85%, and the average micellar size was 66.2 nm. It was demonstrated that the alkaline conditions resulted in a higher release of EGCG than acidic conditions.

A very interesting study was carried out by Picchio et al. [156], who prepared casein micelles crosslinked with glyceraldehyde (GAL) and loaded with Nile red as a model hydrophobic drug. As a function of the dialysis time, the micellar sizes increased from 128 to 509 nm. As expected, by increasing the crosslinking agent concentration, the size of the spherical micelles slightly decreased. The micellar systems with the highest crosslinking degree presented an acceptable loading capacity of 0.44% and an excellent encapsulation efficiency of 89% for the used dye/micelle ratio of 5 μg/mg. The cumulative release of the NR-loaded micelles showed marginal dye leakage at pH = 7.4 but was significantly accelerated by protease and the pH-mediated degradation of the nanocarriers in a dual-responsive fashion.

Gelatin (Gel), the hydrolytic product of collagen, is one of the most versatile natural biopolymers widely used in biomedical applications due to its biocompatibility, biodegradability, and low cost; in addition, the presence of multiple ionizable functional groups in gelatin (–COOH, –NH_2_, phenol, guanidine, imidazole) favors the conjugation of a variety of molecules [157].

**Figure 16:**
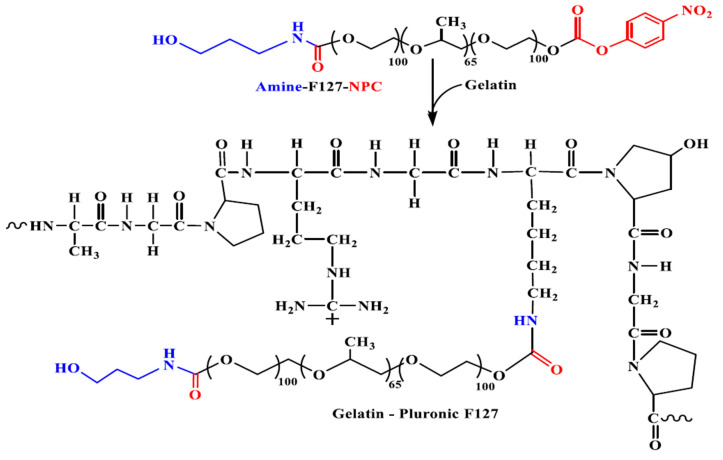
Synthetic scheme of gelatin (Gel)–Pluronic synthesis [161].

Gelatin was modified by a grafting reaction in order to obtain Gel-g-poly(*N*-isopropylacrylamide-co-*N*,*N*-Dimethyl acrylamide-co-10-undecenoic acid)-g-dextran/Fe_3_O_4_ (GPDF) pH-responsive magnetic micelles, which were synthesized in order to encapsulate a hydrophilic insulin promoting factor (nicotinamide) [158]. These spherical micelles had an average diameter of 115.8 nm and a ZP value of −6.35 mV in water. Moreover, the accumulative drug release under pathological pH conditions (pH = 6.6) was found to be three-fold higher than that under normal pH conditions (pH = 7.2). The hydrophilic nature of gelatin was also modified by conjugation with oleylamine using genipin as a crosslinking agent in order to obtain micelles that were loaded with Catechin, as a model antioxidant drug [159]. According to DLS measurements, the average diameter and ZP value of these micelles were 230.6 ± 0.4 nm and −23.4 ± 0.2 mV, respectively, thus proving their colloidal stability at pH = 7.4. Moreover, drug encapsulation efficiency has been found to be higher than 60%. Furthermore, it appeared that these micelles exhibited higher cellular toxicity to MDA-MB-231 cancerous cells than in normal cells (NIH-3T3). Novel glycyrrhetinic acid modified gelatin (GA-Gel) conjugates with three substitution degrees were synthesized and used for the preparation of Dox-loaded micellar systems by an emulsion–solvent evaporation method [160]. The encapsulation efficiency was 63.6–96.2%, and the loading content was 8.3–12.5%. Moreover, these micelles exhibited average diameters in the range of 195 to 235 nm. Furthermore, it appeared that the Dox release exhibited a biphasic behavior in PBS at pH = 7.4 and also that these Dox/GA-Gel micelles could be efficiently accumulated into human liver cancer HepG2 cells. In another study, Gel was modified with two Pluronic copolymers (F87 and F127) (Figure 16) in order to prepare Cur-loaded micelles [161]. Under the impact of dual hydrophobic and electrostatic interactions, these Cur-loaded core–shell micelles had average diameters between 40 and 100 nm. The in vitro release profiles showed a sustainable behavior of Cur and that these micellar systems exhibited higher inhibitory activity against cancer cells growth than that of free Cur.

A series of poly(l-lactide)-grafted gelatin (Gel-g-PLLA) copolymers were synthesized in order to prepare PTX-loaded micelles by a direct dissolution method [162]. The average diameter of these micelles ranged from 45 to 158 nm as the molar mass of the Gel increased from 7000 to 16,000 g/mol. Moreover, both the drug encapsulation and drug-loading efficiencies increased with decreasing the length of Gel chain, reaching maximum values of 57.7% and 1.14%, respectively. Furthermore, PTX exhibited a burst release of about 70% in the first 24 h.

Zein is a protein from maize considered as prolamine due to its characteristic of being insoluble in water but soluble in ethanol. This unique feature was due to the composition of more than 50% of nonpolar amino acids such as alanine, valine and phenylalanine. There are four types of zein, α (19 and 22 kDa), β (14 kDa), γ (16 and 27 kDa) and δ (10 kDa) based on the solubility. Of these four types of zein, α-zein has the highest solubility in alcohol and also major zein in maize, so it is most widely used [163].

Hydrophobic zein was modified with PEG in order to obtain amphiphilic molecules that self-assembled into micelles with sizes in the range of 95 to 125 nm and a ZP of around −7 mV [164]. These micelles were loaded with Cur, and it was shown that the Cur solubility in water was enhanced by 1000–2000 folds. Curcumin-loaded PEG–zein micelles, with an encapsulation efficiency of 95%, showed a sustained drug release and a significantly higher cell uptake than free curcumin in drug-resistant NCI/ADR-RES cancer cells in vitro. Song et al. [165] investigated the same system and reached similar conclusions.

Hydrophobic zein was also modified with hydrophilic lactoferrin (Lf), leading to the formation of amphiphilic copolymers [166]. The obtained spherical micelles were crosslinked with GA in order to increase the stability. Two hydrophobic drugs, rapamycin (RAP) and wogonin (WOG), were loaded with high encapsulation efficiencies. The diameter of the WOG-loaded zein-Lf micelles was 278.6 ± 7.57 nm with a remarkable high positive ZP value (41.6 mV) while RAP-loaded micelles showed a size of 261.6 ± 16.0 nm and a ZP value of 39.4 mV. The crosslinking step in the presence of GA reduced both the micellar diameters and ZP values. The WOG release from the micelles was biphasic and characterized by an initial fast release of about 64% of drug during the first 6 h followed by a second phase of very slow release with about 67.59% of WOG was released after 24 h. On the contrary, RAP showed very slow release from crosslinked dual drug-loaded zein-Lf micelles (˂20% drug release after 72 h) without a considerable initial burst effect. These micellar systems maximized the synergistic cytotoxicity of RAP and WOG in terms of tumor inhibition in MCF-7 breast cancer cells and Ehrlich ascites tumor animal model as a result of enhanced active targeting. The same research group has also used zein-Lf copolymers for the preparation of dasatinib (DAS)-loaded magnetic micelles [167]. This system showed good in vitro serum stability and hemocompatibility accompanied with a sustained release of DAS in acidic pH. In addition, these drug-loaded micelles exhibited increase in vitro cytotoxicity against triple-negative human breast cancer cell line (MDA-MB-231) using an external magnetic field.

## 7. Conclusions

Natural biopolymer-based micellar DDS are high-value biomaterials combining the advantages of both micelles and natural biopolymers, since they have good compatibility, biodegradability, long blood circulation time, non-toxicity, and non-immunogenicity, can load a high amount of drug, and be biodegraded to non-toxic products that are easily assimilated by the human body. Therefore, these micellar systems are regarded as ideal carriers for the delivery of active principles.

However, there are still many challenges related to production, quality control, storage, safety, and selectivity of natural biopolymers-based DDS. In fact, the polydispersities in molecular weight, composition, and structure of these polysaccharides or protein-based amphiphilic graft copolymers are very often incompletely taken into account. The lack of reproducibility of the molecular characteristics of the amphiphilic graft copolymers used for the preparation of drug-loaded micelles depends to a large extent on the inherent composition of the starting natural biopolymers. Moreover, the micellar characteristics of the drug-loaded systems, such as size, polydispersity, morphology, and drug-loading efficiency, are influenced by the drug-loading method. In several studies, alteration of the micelles sizes after drug encapsulation and even of the micellar morphology were noticed, and these modifications could have an impact on the drug release kinetics. Concerning the drug encapsulation, it is suitable to have some interactions (hydrophobic or H-bonds) identifiable by FTIR between the loaded drug and polymeric matrix in order to obtain a sustained and controlled drug release. In fact, in the case of micelles based on amphiphilic biopolymers, no studies have been performed concerning the influence of the hydrophobic sequence length on both the drug encapsulation and release. The majority of these biopolymers were hydrophobically modified only using relatively low molecular weight substances.

From the reviewed studies, it appeared that dialysis is the predominant method for the preparation of drug-loaded micelles. Nevertheless, the range of loaded drug should be extended as almost exclusively model drugs were loaded until now in these micelles. Furthermore, the cellular uptake and drug permeability can be enhanced using ligands for an active targeting drug delivery.

In a very large extent, the polysaccharide or protein-based amphiphilic copolymers studied until now for the preparation of micellar DDS were of graft type and only in a very scarce number of studies were block copolymers investigated. As a perspective, it should be of interest to use oligosaccharides for the preparation of better-defined amphiphilic block copolymers and to perform comparative studies between graft and block copolymers micelles based on biopolymers regarding drug loading and encapsulation efficiencies or micellar morphologies.

For a deeper characterization of the drug-loaded micellar systems, more advanced techniques such as small-angle X-ray or neutron scattering (SAXS and SANS) or NMR analysis should be used as a well-characterized system, from a physicochemical point of view, will facilitate the analysis of his biological behavior.

Another important aspect of the drug-loaded micellar systems is related to their clinical use. Even if until now, only a few micellar DDS met the clinical demands and have been approved for clinical applications, there is a promising future for the extensive in vivo human use of these natural biopolymers as DDS.

## Figures and Tables

**Table 1:** Micellar DDS based on drug/biopolymer conjugates.

Biopolymer	Conjugated Drug	Micellar Size (nm)	ZP Value (mV)	Preparation Method	Reference
*N*-octyl-*N* arginine chitosan ^(+)^	Insulin ^(−)^	257 ÷ 327	4.61 ÷ 6.53	Ionic complexation	[16]
Adipic dihydrazole hyaluronic acid	Quercetin	172.1	−20.3	Direct dissolution	[17]
Hyaluronic acid	Curcumin	84 ÷ 131	−19.43 ÷ 24.27	Sonication	[18]
Hyaluronic acid	Curcumin	209 ÷ 926	−25 ÷ −35	Direct dissolution	[19]
Dithiodipropionic acid hyaluronic acid	Quercetin + Curcumin	172.6	−33.71	Dialysis	[20]
Heparin-graft-deocycholate	Doxorubicin	154.7 166.0	−32.7 −36.7	Sonication	[21]
Pegylated heparin	Pyropheophorbide-a	97.31	−14.63	Not indicated	[22]
Alginate	Curcumin	459	−45	Direct dissolution	[23]
Galactosylated alginate	Curcumin	235	−29	Direct dissolution	[24]
Alginate	Curcumin	200	−53	Direct dissolution	[25,26]
Dextran	Curcumin	222	−15.2	Direct dissolution	[27]
Dextran	Indomethacin	68.3	n.d.	Dialysis	[28]
Dextran	Indomethacin	200	−1.8	Dialysis	[29]
Hydroxyethyl starch	Curcumin	<100	−27.8	Precipitation/dialysis	[30]
Fructose	Curcumin	100 ÷ 150	-	Solution mixing	[31]
Bovine serum albumin functionalized poly(oligo (ethylene glycol) methyl ether methacrylate)	Sprouty 1 (C-12) (Spry1) protein	15 ÷ 25	−9 ÷ 2	Direct dissolution	[32]
Human serum albumin	Adriamycin	100	5 ÷ 10	Thin film hydration	[33]
ABD035 peptide	Paclitaxel	29	8.7	Dialysis	[34]

^(+)^—cation, ^(−)^—anion, n.d.—not determined.

**Table 2:** Characterization techniques and main characteristics of drug-loaded micellar systems.

Characterization Technique	Characteristic
Dynamic and static light scattering (DLS and SLS)	Size Polydispersity Aggregation number N_agg_
Atomic force microscopy (AFM), scanning and transmission electronic microscopy (SEM and TEM, cryo-TEM)	Morphology Size
Fluorescence and surface tension	Critical micellar and association concentrations (CAC and C.M.C.)
Electrophoretic Light Scattering (ELS)	Surface charge (zeta potential ZP)
Differential scanning calorimetry (DSC), X-ray diffraction, Fourier transform infrared spectroscopy (FTIR), Nuclear magnetic resonance (NMR)	Critical micellization temperature (CMT) Degree of crystallinityDrug/polymer interactions
UV spectroscopy, High performance liquid chromatography (HPLC), Liquid chromatography-Mass spectroscopy analysis	Drug loading efficiency (DLE)Drug encapsulation efficiency (DEE)Release kinetics
Small angle X-ray and neutron scattering (SAXS), (SANS)	Size Structural properties

**Table 3:** Drug delivery systems based on miscellaneous polysaccharides.

Copolymer	Drug	Size (nm)	ZP (mV)	Preparation Method	Reference
Xanthan–graft–C16 alkyl chain	Glibenclamide	652.8	−27.6	Precipitation/evaporation	[99]
Pullulan–graft–PCL	ciprofloxacin	142	-	Dialysis	[100]
Pullulan–graft–α-tocopherol	CPT	171.5 ÷ 257.7	-	Dialysis	[101]
Pullulan–graft-SA	Dox	188.75	17.83	Dialysis	[102]
Pullulan–graft–retinoic acid+biotin	Dox	191	−9.45	Direct dissolution	[103]
Pullulan–graft–desaxycholic acid–graft–PEI	Dox DNA	160.8	28	Dialysis	[104]
Pulullan–graft–retinoic acid	Dox	124.5	−3.65	Direct dissolution	[105]
Pullulan–graft–(dibuthyl amino) propyl carbamate	DNA	246.3 ÷ 1214	24.7 ÷ 47	Dialysis	[106]
Pullulan–graft–cholesterol	methatrexate	97.8 144.4 178.0	4.29 3.392.56	Dialysis	[107]
Heparin–graft–β-sitosterol cysteamine	Dox	93.3	−41.8	Sonication and dialysis	[108]
Heparin–graft–α-tocopherol	Docetaxel	139.7	−24.6	Dialysis	[109]
Chitin–graft–hexadecyl	Dox	332.4 ÷ 385.0	38.2 ÷ 44.8	Dialysis	[110]
Guar gum galactomannan–graft–acetic anhydride	Cur	113.9 ÷ 152.8	−14.8 ÷ −18.1	Precipitation/solvent diffusion	[111]
Bletilla striata–graft–SA	Dox	124.17 ÷ 145.93	−13.17 ÷ −18.97	Dialysis	[112]
Phthalated cashew gum	Benznidazole	288.8	−31.8	Precipitation	[113]
Schizophyllan–graft–SA	PTX	156 ÷ 175	−28 ÷ 35.14	Sonication	[114]
Fucoidan–graft–octenyl succinic anhydride	Cur	103.9	−43.57	Not indicated	[115]
Poly(1-*O*-methacryloyl-β-d-fructopyranose)-block-poly(methyl methacylate)	PTX	25 ÷ 46	−9.9 ÷ −22.3	Dialysis	[116]

**Table 4:** Physicochemical properties of different proteins.

Protein	Source	Composition	Molecular Weight
Gelatin	Collagen	4-hydroxylysine, hydroxyproline, glycine, alanine, and proline	15–250 KDa
Albumin	Plasma protein	Single polypeptide with 585 amino acids	66 KDa
Whey protein	Milk protein	α-lactoglobulin (LG), β-LG, lactalbumin, immunoglobulin, lactoferrin	β-LG-18 KDa α-LG-14 KDa
Casein	Milk protein	Proline- α s1, α s2, β and k subunits	α s1-23 KDa α s2-25 KDa β-24 KDa k-19 KDa
Zein	Zea mays L.	High proportion of glutamine and proline	α-zein-22–24 KDa, β-zein-44 KDa, γ-zein-14 KDa
Gliadin	Wheat flour	Glutamine, Proline	28–55 KDa

## Data Availability

No new data were created or analyzed in this study. Data sharing is not applicable to this article.
